# Supplementary material for: Characterization of Two Aldehyde Oxidases from the Greater Wax Moth, Galleria mellonella Linnaeus. (Lepidoptera: Pyralidae) with Potential Role as Odorant-Degrading Enzymes
Source: Insects. 2022 Dec 12;13(12):1143. doi: 10.3390/insects13121143 (PMC9782417; doi:10.3390/insects13121143)
Supplement: Supplementary file 1 [file insects-13-01143-s001.zip › References Table S1.pdf]

References Table S1

83. Radovic, B.S.; Careri, M.; Mangia, A.; Musci, M.; Gerboles, M.; Anklam, E. Contribution of dynamic headspace GC-MS analysis of aroma compounds to authenticity testing of honey. *Food Chem*. 2001; 72, 511-520. https://doi.org/10.1016/S0308-8146(00)00263-6.
84. Pino, J.; Marbot, R.; Delgado, A.; Zumárraga, C.; Sauri, E. Volatile constituents of propolis from honey bees and stingless bees from Yucatán. *J. Essent. Oil Res*. 2006; 18, 53-56. https://doi.org/10.1080/10412905.2006.9699384.
85. Choi, H.S. Character impact odorants of *Citrus hallabong* [(C. unshiu Marcov x C. sinensis Osbeck) x C. reticulata Blanco] Cold-Pressed Peel Oil. *J. Agric. Food. Chem*. 2003; 51, 2687-2692. https://doi.org/10.1021/jf021069o.
86. Torto, B.; Suazo, A.; Alborn, H.; Tumlinson, J.; Teal, P. Response of the small beetle (*Aethina tumida*) to a blend of chemicals identified from honeybee (*Apis mellifera*) volatiles. *Apidologie*. 2005; 36, 523-532. https://doi.org/10.1051/apido:2005038.
87. Panseri, S.; Manzo, A.; Chiesa, L.; Giorgi, A. Melissopalynological and volatile compounds analysis of buckwheat honey from different geographical origins and their role in botanical determination. *J. Chem.* 2013; 904202, 1-11. https://doi.org/10.1155/2013/904202.
88. Adams, R.P. The leaf essential oils and chemotaxonomy of *Juniperus* sect. *Juniperus*. *Biochem. System. Ecol*. 1998; 637-645. https://doi.org/10.1016/S0305-1978(98)00020-9.
89. Ferber, C.E.; Nursten, H.E. The aroma of beeswax. *J. Sci. Food Agric.* 1977; 28, 511-518. https://doi.org/10.1002/jsfa.2740280608.
90. Pattamayutanon, P.; Angeli, S.; Thakeow, P.; Abraham, J.; Disayathanoowat, T.; Chantawannakul, P. Volatile organic compounds of Thai honeys produced from several floral sources by different honey bee species. *PLoS ONE*. 2017; 12, e0172099. https://doi.org/10.1371/journal.pone.0172099.
91. Karioti, A.; Skaltsa, H.; Demetzos, C.; Perdetzoglou, D.; Economakis, C.D.; Salem, A.B. Effect of nitrogen concentration of the nutrient solution on the volatile constituents of leaves of *Salvia fruticosa* Mill. in solution culture. *J. Agric. Food Chem*. 2003; 51, 6505-6508. https://doi.org/10.1021/jf030308k.
92. Kaskoniene, V.; Venskutonis, P. Floral markers in honey of various botanical and geographic origins: A review. *Compr. Rev. Food Sci. Food Saf*. 2010; 9, 620-634. https://doi.org/10.1111/j.1541-4337.2010.00130.x.
93. Hamm, S.; Bleton, J.; Connan, J.; Tchapla, A. A chemical investigation by headspace SPME and GC-MS of volatile and semi-volatile terpenes in various olibanum samples. *Phytochemistry*. 2005; 66, 1499-1514. https://doi.org/10.1016/j.phytochem.2005.04.025.
94. Skaltsa, H.D.; Demetzos, C.; Lazari, D.; Sokovic, M. Essential oil analysis and antimicrobial activity of eight Stachys species from Greece. *Phytochemstry*. 2003; 64, 743-752. https://doi.org/10.1016/s0031-9422(03)00386-8.
95. De Kraker, J.W.; Schurink, M.; Franssen, M.C.; König, W.A.: De Groot, A.; Bouwmeester, H.J. Hydroxylation of sesquiterpenes by enzymes from chicory (*Cichorium intybus* L.) roots. *Tetrahedron.* 2003; 59, 409-418. https://doi.org/10.1016/S0040-4020(02)01479-5.
96. Tellez, M.R.; Canel, C.; Rimando, A.M.; Duke, S.O. Differential accumulation of isoprenoids in glanded and glandless *Artemisia annua* L. Phytochemistry. 1999; 52, 1035-1040. https://doi.org/10.1016/S0031-9422(99)00308-8.
97. Schmitt, T.; Herzner, G.; Weckerle, B.; Schreier, P.; Strohm, E. Volatiles of foraging honeybees *Apis mellifera* (Hymenoptera: Apidae) and their potential role as semiochemicals. *Apidologie*. 2007; 38, 164-170. https://doi.org/10.1051/apido:2006067.
98. Apel, M.A.; Sobral, M.; Schapoval, E.E.; Henriques, A.T.; Menut, C.; Bessiere, J.M. Essential oil composition of *Eugenia florida* and *Eugenia mansoi*. *J. Essent. Oil Res.* 2004. 16, 321-322. https://doi.org/10.1080/10412905.2004.9698732.
99. Zahn, D.K.; Moreira, J.A.; Millar, J.G. Identification, synthesis, and bioassay of a male-specific aggregation pheromone from the harlequin bug, *Murgantia histrionica*. *J. Chem. Ecol*. 2008; 34, 238-251. https://doi.org/10.1007/s10886-007-9415-x.
100. Jordán, M.J.; Margaria, C.A.; Shaw, P.E.; Goodner, K.L. Volatile components and aroma active compounds in aqueous essence and fresh pink Guava fruit puree (*Psidium guajava* L.) by GC-MS and multidimensional GC/GC-O. *J. Agric. Food Chem*. 2003; 51, 1421-1426. https://doi.org/10.1021/jf020765l.
101. Abd El-Wahed, A.A.; Khalifa, S.A.M.; Sheikh, B.Y.; Farag, M.A.; Saeed, A.; Larik, F.A.; Koca-Caliskan, U.; AlAjmi, M.F.; Hassan, M.; Wahabi, H.A.; et al. Bee Venom Composition: From Chemistry to Biological Activity. Studies in Natural Products Chemistry (ed. A. Rahman), 2019; pp. 459-484. Elsevier.
102. Sotomayor, J.A.; Martinez, R.M.; Garcia, A.J.; Jordan, M.J. *Thymus zygis* subsp. Gracilis: Watering level effect on phytomass production and essential oil quality. *J. Agric. Food Chem.* 2004; 52, 5418-5424. https://doi.org/10.1021/jf0496245.
103. Tepe, B.; Donmez, E.; Unlu, M.; Candan, F.; Daferera, D.; Vardar-Unlu, G.; Polissiou, M.; Sokmen, A. Antimicrobial and antioxidative activities of the essential oils and methanol extracts of *Salvia cryptantha* (Montbret et Aucher ex Benth.) and *Salvia multicaulis* (Vahl). *Food Chem*. 2004. 84, 519-525. https://doi.org/10.1016/S0308-8146(03)00267-X.
104. Adams, R.P.; Dev, V. Synthesis and GC-MS analysis of angelates and tiglates as an aid to identification of these components in essential oils. *Flavour Fragr. J.* 2010; 25, 71-74. https://doi.org/10.1002/ffj.1968.
105. Ali, N.A.; Wurster, M.; Arnold, N.; Teicher, A.; Schmidt, J.; Lindequist, U.; Wessjohann, L. Chemical composition and biological activity of essential oils from the Oleogum resins of three endemic soqotraen Boswellia species. *Rec. Nat. Prod*. 2008; 2, 6-12.

106. Lima-Neto, J.; Lopes, J.A.; Moita-Neto, J.M.; Lima, S.G.; Luz, C.F.; Citó, A.M. Volatile compounds and palynological analysis from pollen pots of stingless bees from the mid-north region of Brazil. Braz. *J. Pharm. Sci.* 2017; 53, 1-9. https://doi.org/10.1590/s2175-97902017000214093.
107. Demyttenaere, J.C.; Sánchez-Martínez, J.I.; Verhé, R.; Sandra, P.; De Kimpe, N. Analysis of volatiles of malt whisky by solid-phase microextraction and stir bar sorptive extraction. *J. Chromatogr. A.* 2003; 985, 221-232. https://doi.org/10.1016/s0021-9673(02)01471-1.
108. El-Sayed, A.M.; Heppelthwaite, V.J.; Manning, L.M.; Gibb, A.R.; Suckling, D.M. Volatile constituents of fermented sugar baits and their attraction to lepidopteran species. *J. Agric. Food Chem.* 2005; 53, 953-958. https://doi.org/10.1021/jf048521j.
109. Wang, Q.; Yang, Y.; Zhao, X.; Zhu, B.; Nan, P.; Zhao, J.; Wang, L.; Chen, F.; Liu, Z.,; Zhong, Y. Chemical variation in the essential oil of *Ephedra sinica* from Northeastern China. *Food Chem*. 2006; 98, 52-58. https://doi.org/10.1016/j.foodchem.2005.04.033.
110. McDaniel, C.A.; Schmidt, J.O.; Howard, R.W. Mandibular gland secretions of the male beewolves *Philanthus crabroniformis*, *P. barbatus*, and *P. pulcher* (Hymenoptera: Sphecidae). *J. Chem. Ecol.* 1992; 18, 27-37. https://doi.org/10.1007/BF00997162.
111. Marques, F.; McElfresh, J.S.; Millar, J. Kováts Retention Indexes of Monounsaturated C_{12}, C_{14}, and C16 Alcohols, Acetates and Aldehydes Commonly Found in Lepidopteran Pheromone Blends. *J. Braz. Chem. Soc.* 2000; 11, 592-599. https://doi.org/10.1590/s0103-50532000000600007.
112. De Simon, B.F.; Estruelas, E.; Munoz, A.M.; Cadahia, E.; Sanz, M. Volatile compounds in acacia, chestnut, cherry, ash, and oak woods, with a view to their use in cooperage. *J. Agri. Food Chem.* 2009. 57, 3217-3227. https://doi.org/10.1021/jf803463h.
113. Witte, L.; Rubiolo, P.; Bicchi, C.; Hartmann, T. Comparative analysis of pyrrolizidine alkaloids from natural sources by gas chromatography-mass spectrometry. *Phytochemistry*. 1993. 32, 187-196. https://doi.org/10.1016/0031-9422(92)80130-7.
114. DeGrandi-Hoffman, G.; Chambers, M.; Hooper, J.; Schneider, S. Description of an intermorph between a worker and queen in african honey bees *Apis mellifera* scutellata (Hymenoptera: Apidae). *Ann. Entomol. Soc. Am.* 2004; 97, 1299-1305. https://doi.org/10.1603/0013-8746(2004)097[1299:DOAIBA]2.0.CO;2.
115. Zhao, Y.; Li, J.; Xu, Y.; Duan, H.; Fan, W.; Zhao, G. Extraction, preparation and identification of volatile compounds in Changyu XO Brandy. *Chin. J. Chromatogr*. 2008. 26, 212-222. https://doi.org/10.1016/S1872-2059(08)60014-0.
116. Morteza-Semnani, K.; Akbarzadeh, M.; Moshiri, K. The essential oil composition of *Eupatorium cannabinum* L. from Iran. *Flavour Fragr. J.* 2006; 21, 521-523. https://doi.org/10.1002/ffj.1687.
117. Gómez, E.; Ledbetter, C.A.; Hartsell, P.L. Volatile compounds in apricot, plum, and their interspecific hybrids. *J. Agric. Food Chem.* 1993; 41,1669-1676. https://doi.org/10.1021/jf00034a029.
118. Suwannapong, G.; Benbow, M.; Chinokul, C.; Seanbualuang, P.; Sivaram, V. Bioassay of the mandibular gland pheromones of *Apis florea* on the foraging activity of dwarf honey bees. *J. Apic. Res.* 2011; 50, 212-217. https://doi.org/10.3896/IBRA.1.50.3.05.
119. Leffingwell, J.C.; Alford, E.D. Volatile constituents of perique tobacco. Elec. *J. Env. Agricult. Food Chem.* 2005; 4, 899-915. http://www.leffingwell.com/download/Volatile%20Constituents%20of%20Perique%20Tobacco4.pdf.
120. Rostad, C.E.; Pereira, W.E. Kovats and Lee Retention Indices determined by Gas Chromatography/Mass Spectrometry for organic compounds of environmental interest. *J. High. Resolut Chromatogr Chromatogr Commun*. 1986; 9, 328-334. https://doi.org/10.1002/JHRC.1240090603.
121. Jaramillo, J.; Torto, B.; Mwenda, D.; Troeger, A.; Borgemeister, C.; Poehling, H.M.; Francke, W. Coffee berry borer joins bark beetles in coffee klatch. *PLoS ONE*. 2013; 8, e74277. https://doi.org/10.1371/journal.pone.0074277.
122. Harrison, B.M.; Priest, F.G. Composition of peaks used in the preparation of malt for Scotch Whisky production - influence of geographical source and extraction depth. *J. Agric. Food Chem*. 2009; 57, 2385-2391. https://doi.org/10.1021/jf803556y.
123. Graham, J. The attraction of bumble bee (Hymenoptera: Apidae, *Bombus impatiens* Cresson) colonies to small hive beetles (Coleoptera: Nitidulidae, Aethina tumida Murray). 2009. A thesis presented to the graduate school of the University of Florida. http://ufdc.ufl.edu/UFE0024736/00001.
124. Lai, W.C.; Song, C. Temperature-programmed retention indices for GC. and GC-MS analysis of coal- and petroleum-derived liquid fuels. *Fuel*. 1995; 74, 1436-1451. https://doi.org/10.1016/0016-2361(95)00108-H.
125. Zaikin, V.G.; Borisov, R.S. Chromatographic-mass spectrometric analysis of Fischer-Tropsch synthesis products. *J. Anal. Chem.* 2002; 57, 544-551. https://doi.org/10.1023/A:1015754120136.
126. Jordán, M.J.; Margaría, C.A.; Shaw, P.E.; Goodner, K.L. Aroma active components in aqueous kiwi fruit essence and kiwi fruit puree by GC-MS and multidimensional GC/GC-O. *J. Agric. Food Chem.* 2002; 50, 5386-5390. https://doi.org/10.1021/jf020297f.
127. Karabadagias, I.; Karabadagias, V.; Badeka, A. The honey volatile code: A collective study and extended version. *Foods*. 2019; 8, 508. https://doi.org/10.3390/foods8100508.
128. Pino, J.A.; Mesa, J.; Munoz, Y.; Marti, M.P.; Marbot, R. Volatile components from mango (*Mangifera indica* L.) cultivars. *J. Agric. Food Chem.* 2005; 53, 2213-2223. https://doi.org/10.1021/jf0402633.
129. Kim, K.R.; Kim, H. Gas chromatographic profiling and screening for phenols as isobutoxycarbonyl derivatives in aqueous samples. *J. Chromatogr. A*. 2000; 866, 87-96. https://doi.org/10.1016/s0021-9673(99)01068-7.
